# Molecular Biomarkers Predictive of Sertraline Treatment Response in Young Children With Autism Spectrum Disorder

**DOI:** 10.3389/fgene.2020.00308

**Published:** 2020-04-15

**Authors:** Reem Rafik Alolaby, Poonnada Jiraanont, Blythe Durbin-Johnson, Mittal Jasoliya, Hiu-Tung Tang, Randi Hagerman, Flora Tassone

**Affiliations:** ^1^College of Health Sciences, California Northstate University, Rancho Cordova, CA, United States; ^2^Faculty of Medicine, King Mongkut’s Institute of Technology Ladkrabang, Bangkok, Thailand; ^3^Division of Biostatistics, School of Medicine, University of California, Davis, Davis, CA, United States; ^4^Department of Biochemistry and Molecular Medicine, School of Medicine, University of California, Davis, Davis, CA, United States; ^5^MIND Institute, University of California Davis Medical Center, Davis, Davis, CA, United States; ^6^Department of Pediatrics, School of Medicine, University of California, Davis, Davis, CA, United States

**Keywords:** Autism Spectrum Disorders, serotonin, sertraline, selective serotonin reuptake inhibitor, molecular biomarkers

## Abstract

Sertraline is one among several selective serotonin reuptake inhibitors (SSRIs) that exhibited improvement of language development in Autism Spectrum Disorder (ASD); however, the molecular mechanism has not been elucidated. A double blind, randomized, 6-month, placebo-controlled, clinical trial of low-dose sertraline in children ages (3–6 years) with ASD was conducted at the UC Davis MIND Institute. It aimed at evaluating the efficacy and benefit with respect to early expressive language development and global clinical improvement. This study aimed to identify molecular biomarkers that might be key players in the serotonin pathway and might be predictive of a clinical response to sertraline. Fifty eight subjects with the diagnosis of ASD were randomized to sertraline or placebo. Eight subjects from the sertraline arm and five from the placebo arm discontinued from the study. Furthermore, four subjects did not have a successful blood draw. Hence, genotypes for 41 subjects (20 on placebo and 21 on sertraline) were determined for several genes involved in the serotonin pathway including the serotonin transporter-linked polymorphic region (*5-HTTLPR*), the tryptophan hydroxylase 2 (*TPH2*), and the Brain-Derived Neurotrophic Factor (*BDNF*). In addition, plasma levels of *BDNF*, Matrix metallopeptidase 9 (*MMP-9*) and a selected panel of cytokines were determined at baseline and post-treatment. Intent-to-treat analysis revealed several primary significant correlations between molecular changes and the Mullen Scales of Early Learning (MSEL) and Clinical Global Impression Scale – Improvement (CGI-I) of treatment and control groups but they were not significant after adjustment for multiple testing. Thus, sertraline showed no benefit for treatment of young children with ASD in language development or changes in molecular markers in this study. These results indicate that sertraline may not be beneficial for the treatment of children with ASD; however, further investigation of larger groups as well as longer term follow-up studies are warranted.

## Introduction

According to the American Psychiatric Association’s Diagnostic and Statistical Manual of Mental Disorders, version-5, Autism Spectrum Disorder (ASD) is a neurodevelopmental disorder characterized by impairments in two domains: (1) communication and social interaction and (2) restricted, repetitive, and stereotyped patterns of behaviors and interests (APA, 2013). According to Center for Disease Control and Prevention, 1 in 59 children is diagnosed with ASD. Approximately 30% of individuals with ASD also require psychological and psychiatric treatments for behavioral problems including hyperactivity, impulsivity, inattention, aggression, property destruction, self-injury, mood disorders, psychosis, and tic disorders (Lecavalier, 2006; Butler et al., 2012). Symptoms of ASD usually begin in early childhood and are frequently accompanied by intellectual disability (75%), dysmorphic features and epilepsy (25%), and occasionally MRI and EEG abnormalities (Miles and Hillman, 2000; Brooks-Kayal, 2010; Chugani, 2012). However, since there are no definite biomarkers, the diagnosis is based on a standardized clinical assessment and relies basically on behaviors including speech delay and language deficits (Lord et al., 2018).

Several neuroimaging and genetic studies indicate dysregulation of serotonin in the pathogenesis of ASD. Serotonin is a neurotransmitter synthesized in the central nervous system (CNS) and plays a pivotal role in brain development. Dysregulation in serotonin is associated with aggression, anxiety, mood, impulsivity, sleep, ingestion behavior, and reward systems (Cook and Leventhal, 1996; Chugani, 2002). Perhaps the most intriguing 5HT-related finding in ASD is hyperserotonemia, an increase of platelet 5-HT, which has been consistently observed in about one third of subjects with ASD (Anderson et al., 1990; Cook and Leventhal, 1996; Mulder, 2006; Hranilovic et al., 2007). In addition, reduction in uptake of tryptophan (the precursor of 5-HT) and 5-HT synthesis, decreased 5-HT_2A_ receptor binding, and binding capacity of 5-HT transporter molecules (*SERT, 5-HTT*) have been detected in autistic brain using positron emission tomography and single-photon emission computed tomography (Chugani et al., 1999; Chandana et al., 2005; Makkonen et al., 2008; Goldberg et al., 2009; Oblak et al., 2013).

Evidence showed the global 5-HT synthesis in frontal, temporal, and parietal cortex in children aged 2–5 years with ASD was significantly attenuated compare to neurotypical children which correlated with altered language development suggesting a disruption of serotonergic system in ASD brains during early childhood (Chugani et al., 1999). As mentioned earlier, the serotonin transporter (*5-HTT*) involved in hyperserotonemia is encoded by *SLC6A4* gene, for which a causal link to ASD has been reported (Marazziti et al., 2000; Wassink et al., 2007). The most extensively studied polymorphism associated with ASD is in the serotonin Transporter-linked polymorphic region (*5-HTTLPR*) located within the promoter region of the gene and presented with two alleles designated long (l) and short (s). Both modulate the expression and function of the serotonin transporter and they have been correlated with cerebral gray matter volume, hippocampal volume and amygdala response in ASD (Wassink et al., 2007), aggression and ADHD in males (Cadoret et al., 2003).

In addition, case-control and family-based investigations with molecular approaches unveiled serotonin-associated candidate genes in ASD including tryptophan hydroxylase 2 (*TPH2*), Brain-Derived Neurotrophic Factor (*BDNF*) and Matrix metallopeptidase 9 (*MMP-9*) (Noroozi et al., 2016; Meng et al., 2017; Barrie et al., 2018). *TPH2* is a gene located on chromosome 12 encoding for a rate-limiting enzyme for brain serotonin synthesis playing a role in ASD susceptibility associated phenotypic impairments and repetitive behavior (Coon et al., 2005; McKinney et al., 2005; Zafeiriou et al., 2009). However, some studies have shown no significant correlation between *TPH2* variants and ASD (Ramoz et al., 2006; Sacco et al., 2007).

*BDNF* has long been the focus of attention for underlying mechanisms leading to ASD. Several studies showed significant correlations between elevated *BDNF* levels in serum or blood and ASD (Miyazaki et al., 2004; Nishimura et al., 2007; Correia et al., 2010; Ricci et al., 2013; Zhang et al., 2014). *BDNF* has a trophic effect for dopaminergic neurons both during brain development and maturity. It is involved in regulating neuronal survival, morphology, differentiation, synapse formation, and normal cognitive function. *BDNF* is trophic for CNS serotonin as well (McAllister, 2001; Anderson and Lombroso, 2002; Binder and Scharfman, 2004a, b, Gunstad et al., 2008). A study in rodents has indicated that *BDNF* and serotonin react to the same environmental factors in reciprocal manner (Mattson et al., 2004). For instance, heterozygous *BDNF* mice are extremely deprived of serotonin clearance in the CA3 region of hippocampus due to functional impairment of serotonin transporter rather than total amount of serotonin transporter (Daws et al., 2007). Notably, 5-HT and extracellular matrix (ECM) regulate learning and memory formation through morphological changes of dendritic spines during brain development (Udo et al., 2005; Dityatev et al., 2010).

*MMP-9*, one of various ECM modifiers, serves a pivotal role in long-term memory, synaptic plasticity, development of the CNS during synaptogenesis, as well as in neuro-inflammation, which are features consistently found in children with ASD (Rosenberg, 2002; Yong, 2005; Ethell and Ethell, 2007; Huntley, 2012). Intriguingly, amniotic fluid samples derived from 331 ASD individuals exhibited increased levels of *MMP-9* which may imply the neuroplastic disruption during prenatal period (Abdallah et al., 2012). Elevated *MMP-9* activation can also cause increased *BDNF* release and may contribute to the ASD phenotype, including autistic-like behavior and macrocephaly (Courchesne et al., 2003; Lainhart et al., 2006; Yoo et al., 2016). Taken together, serotonin-associated genes including *TPH2*, *BDNF*, and *MMP-9* may be involved in the disruption of the serotonergic system and ultimately play a role in the pathology of ASD.

Selective serotonin reuptake inhibitors (SSRIs), which are widely prescribed and can influence peripheral and CNS 5-HT levels may correct dysregulation and alleviate ASD symptoms (Kolevzon et al., 2006). The Food and Drug Administration has approved several SSRIs including citalopram, escitalopram, fluoxetine, fluvoxamine, and sertraline for treating psychiatric symptoms in ASD (Nadeau et al., 2011). However, a placebo-controlled study reporting the efficacy of sertraline in ASD patients has not been carried out. Steingard et al. (1997) reported the results of an open-label study of low dosed sertraline (25–50 mg daily) in nine children with ASD (6–12 years) and showed significant improvement in anxiety, irritability, and inflexibility. Later, McDougle et al. (1998) reported marked reduction of aggressive and repetitive behaviors in adult ASD patients.

Thus, evidence suggests that sertraline could be useful in young children with ASD since the serotonergic system is disrupted in early development of ASD children. Based on these findings, a 6-month randomized, placebo-controlled, double-blind clinical trial of low-dose sertraline in children ages 39–71 months old with ASD was conducted at the UC Davis MIND Institute to evaluate the efficacy and benefit with respect to early expressive and receptive language development and global clinical improvement (Potter et al., 2019). In the present study, we further investigated the participants to identify molecular biomarkers predictive of efficacy and responsiveness to sertraline treatment in ASD. Candidate genes were selected specifically on the basis of their role in serotonin metabolism, uptake and transport, including *TPH-2*, *5-HTTLPR*, *MMP-9*, and *BDNF*.

## Materials and Methods

### Study Design

A 6-month randomized, placebo-controlled, double-blind clinical trial of sertraline treatment was conducted at the UC Davis MIND Institute. 179 subjects were screened for eligibility, and a total of 58 were randomized; 32 subjects to sertraline and 26 to placebo. Thirteen subjects, eight from the sertraline arm and five from the placebo arm, discontinued from the study. Forty five ASD subjects aged 39–71 months completed the sertraline clinical trial. However, for four subjects a blood sample was not obtained. Thus, biological specimens were collected for 41 subjects including eight females and 33 males. All patients were randomized and either received a placebo (*n* = 20) or sertraline (*n* = 21). This was the first exposure to sertraline for all the children. Sertraline was administered in liquid form in a dose of 2.5 mg per day in patients ages 2–3 years and 5.0 mg per day in those 4–6 years. The amount that was used at each 3 months visit was measured to document compliance. More details are described in Potter et al. (2019). Biological samples collected at baseline and post-treatment were approved by the UC Davis Institutional Review Board.

### Clinical Measures

Clinical assessment of study participants involved primary outcome measures: Mullen Scales of Early Learning (MSEL) (Mullen, 1995) expressive language raw score, expressive language standard score and Clinical Global Impression Scale-Improvement (CGI-I). The CGI-I score is a follow-up measure scored as follows: 1 = very much improved since the initiation of treatment; 2 = much improved; 3 = minimally improved; 4 = no change from baseline; 5 = minimally worse; 6 = much worse; 7 = very much worse since the initiation of treatment (Busner and Targum, 2007). Additionally, the following secondary outcome measures were used: MSEL subscales: fine motor, visual reception and receptive language score. For each participant, all assessments were completed both at baseline and at the 6-month follow up visit.

### Molecular Measures

Genomic DNA was isolated from 3 ml of peripheral blood following standard procedure (Qiagen, Valencia, CA) and used for genotype analysis. Plasma was collected using EDTA containing blood collection tubes: blood was centrifugated for 10 min at 1000 × *g* within 2 h of blood collection. Plasma was collected, aliquoted, and stored at −80°C.

*5-HTTLPR* was performed using 100–200 ng genomic DNA and 20 μM of the following specific primers, forward HTTP2A (5′-TGA ATG CCA GCA CCT AAC CC-3′), reverse HTTP2A (5′-TTC TGG TGC CAC CTA GAC GC-3′), following PCR conditions as detailed in Tassone et al. (2011). *BDNF* (rs6265) and *TPH2* (rs4290270, rs7305115, rs11178997, and rs4570625) genotypes were determined using TaqMan SNP Genotyping Assay (Applied Biosystems) and the 7900HT Sequencer and Sequence Detection System Software (Applied Biosystems, Inc., Foster City, CA).

Plasma samples were collected from purple top EDTA containing collection tubes, to compare chemokines, *MMP-9* and *BDNF* levels before and after intervention. Furthermore, plasma samples from nine typically developing male controls (Age range: 4–18 years old) were used to compare their *BDNF* and *MMP-9* plasma levels to children with ASD, respectively.

To determine the *MMP-9* plasma activity, the Human MMP Magnetic Bead Panel 2 96-Well Plate Assay (Merck Millipore, Billerica, MA) was used. Preparation of plasma samples and reactions were performed according to the manufacturer’s protocol.

*BDNF* plasma levels were measured using a Milliplex assay (EMD-Millipore-Bellerica/MA). Samples were diluted 100-fold with Assay Buffer. Overnight incubation was carried out for 17 h at 4°C with shaking. Samples were measured within 1 h of finishing protocol using Luminex bead reader.

Cytokine and Chemokine levels were measured using Milliplex MAP Human Cytokine and Chemokine Magnetic Bead Panel Immunoassay (EMD-Millipore-Bellerica/MA). It was a 10 plex kit which included beads specific for IL-1b, IL-2, IL-4, IL-5, IL-6, IL-10, IL-12(p70), IL-13, IFNg, TNFa. Plasma samples were prepared according to manufacturer’s protocol and the plates were read on Bio-Plex 200 System (Bio-Rad).

### Statistical Analysis

Mean subject age was compared between treatment groups using a two-sample *t*-test, and the proportions of male and female subjects were compared between treatment groups using Fisher’s Exact Test.

The association between MSEL receptive and expressive language raw scores at baseline and molecular measures at baseline was analyzed using linear regression. The association between baseline CGI-S score and molecular measures at baseline was analyzed using proportional odds logistic regression (Agresti and Kateri, 2011). *P*-values were adjusted for multiple testing across molecular measures using the Benjamini-Hochberg false discovery rate controlling method (Benjamini and Hochberg, 1995).

The association between *BDNF* expression at baseline and *BDNF* genotype was analyzed using a one-way ANOVA model and Tukey HSD pairwise comparisons.

The changes in treatment group subjects in MSEL receptive language and expressive language scores were compared between genotypes using one-way ANOVA models and Tukey pairwise comparisons. CGI-I scores were compared between genotypes using proportional odds logistic regression.

Changes from baseline in molecular measures were compared between groups using one-way ANOVA models, with *P*-values adjusted across molecular measures using the Benjamin-Hochberg method.

The association between changes from baseline in MSEL receptive language and expressive language and changes from baseline in molecular measures were analyzed by group using linear models with effects for the molecular measure, treatment group, and their interaction. The association between CGI-I and changes from baseline in molecular measures was analyzed using proportional odds logistic regression models with effects for the molecular measure, treatment group, and their interaction.

Baseline expression of *BDNF* and *MMP-9* was compared between cases and controls using ANOVA models.

*BDNF* was log transformed prior to analysis for scaling purposes and to more closely satisfy ANOVA model assumptions. *MMP-9* was log transformed in the analysis in which it was used as a response (that comparing *MMP-9* between cases and controls) in order to more closely satisfy model assumptions.

Analyses were conducted using R, version 3.5.3 (R Core Team, 2019) Proportional odds logistic regression models were fitted using the R package ordinal, version 2019.2-9 (Christensen, 2019).

## Results

### Study Subjects

Biological samples were collected at baseline for 45 subjects (pre-treatment), among which 41 subjects had their follow up visits (post treatment). Forty one biological samples were used to compare the plasma levels of *MMP-9*, *BDNF* and selected cytokines. Among the 41 subjects, 20 were on placebo and 21 were treated with sertraline. The mean age at baseline for the placebo group was 51.9 months and that of the treatment group was 50.6 months (Table 1).

**TABLE 1:** Subject Demographic Characteristics. Summary of age and gender by treatment group.

		Placebo	Treatment	All subjects	*P*-value
*Age^a^ at*	N^c^	20	21	41	0.71
*baseline*	Mean (SD)	51.9 (10.5)	50.6 (11.4)	51.2 (10.9)	
*(months)*	Median (range)	54.5 (31–71)	54 (32–69)	54 (31–71)	
*Gender*^b^	Female	4 (20%)	4 (19%)	8 (19.5%)	1
	Male	16 (80%)	17 (81%)	33 (80.5%)	

*^a^Age was compared between groups using a two-sample t-test. ^b^Gender was compared between groups using Fisher’s exact test. ^c^N indicates the number of subjects.*

Plasma samples derived from age and gender matched control children were utilized for measuring *BDNF* and *MMP-9* levels for comparison.

### Molecular Measures

Linear regression analyses of MSEL receptive language raw score at baseline by molecular measures at baseline showed that higher expression of IL-5 at baseline is associated with a significantly lower baseline MSEL receptive language raw score (*P* = 0.030), however, this result was no longer significant after multiple testing adjustment (adjusted *P* = 0.362).

**FIGURE 1:**
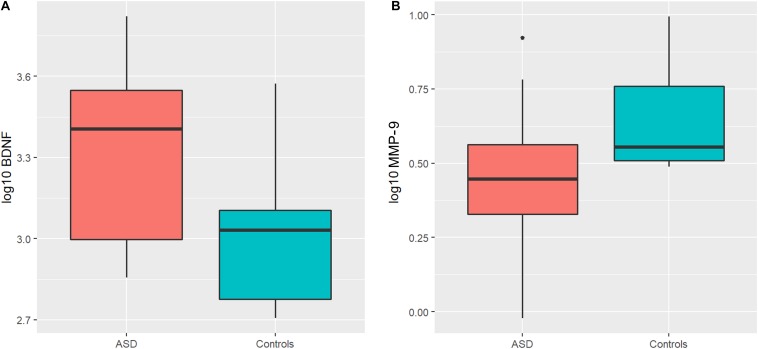
Boxplots showing significantly higher plasma *BDNF* levels **(A)** and lower *MMP-9* levels **(B)** in the ASD group compared to controls. The heavy line in each box represents the median, the lower and upper box edges represent the 25th and 75th percentiles, respectively, and the lower and upper whiskers represent the smallest and largest observations, respectively.

An ANOVA model was used to compare *BDNF* expression level between children with ASD and controls. The results show that children with ASD had significantly higher *BDNF* expression levels compared to typical age matched children (Figure 1A). However, sertraline did not normalize the *BDNF* levels in those treated with sertraline compared to the placebo group. Lower *MMP-9* expression levels were observed children with ASD than controls (*P* = 0.026) (Figure 1B). No difference in *BDNF* and *MMP-9* expression were observed between the treatment and the placebo group at baseline.

When comparing the changes in MSEL expressive language raw score by *TPH2* (rs11178997) (A/T), we observed that subjects with the AT genotype had significantly higher MSEL expression language raw scores than subjects with the TT genotype (*P* = 0.031) (Table 2). Furthermore, after comparing the changes in MSEL expressive language raw scores by TPH2 (rs4290270), it was shown that subjects with the AA genotype had marginally significantly higher changes in MSEL expressive language raw scores compared to subjects with the TT genotype (0.049), with subjects with the AA genotype showing an increase in score and subjects with the TT genotype showing a decrease in score (Table 2).

**TABLE 2:** MSEL Expressive Language Raw Score – Change by TPH2 (rs11178997) (A/T) and TPH2 (rs4290270) (A/T) genotypes in the treatment group.

	Genotype^a^	Mean (95% Confidence Interval)	*P*-value^b^
**TPH2**	AT (*n*^c^ = 3)	16.00 (4.90, 27.10)	0.008
**(rs11178997)**	TT (*n*^c^ = 12)	2.08 (−3.46, 7.63)	0.432
**TPH2**	AA (*n*^c^ = 6)	12.0 (4.47, 19.53)	0.004
**(rs4290270)**	AT (*n*^c^ = 8)	0.5 (−6.02, 7.02)	0.872
	TT (*n*^c^ = 3)	−4.0 (−14.65, 6.65)	0.434

	**Comparison**	**Difference in means (95% Confidence Interval)**	***P*-value**^b^

**TPH2 (rs11178997)**	AT – TT	13.9 (1.51, 26.3)	0.031
**TPH2**	AA – AT	11.5 (−0.6593, 23.7)	0.065
**(rs4290270)**	AA – TT	16.0 (0.0797, 31.9)	0.049
	AT – TT	4.5 (−10.7425, 19.7)	0.725

*^a^Genotypes with fewer than three subjects with non-missing data were excluded. ^b^P-values are adjusted for multiple pairwise comparisons using the Tukey HSD method. ^c^N indicates the number of subjects.*

**FIGURE 2:**
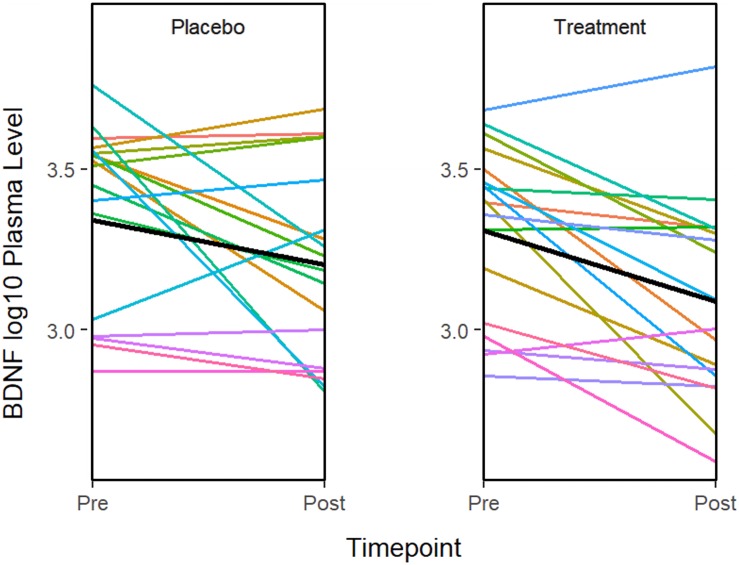
The graphs show a significant improvement for baseline to after treatment in the BDNF plasma level in both the sertraline and placebo groups. A line plot of log10 BDNF plasma levels by time point. Colored lines represent individual subjects and the heavy black lines show the group means.

ANOVA models were used to compare changes in molecular measures between treatment groups. The results showed that *BDNF* expression decreases significantly in both the treatment (*P* = 0.001) and placebo (*P* = 0.011) groups (Figure 2). Although in the treatment group, IL-5 (*P* = 0.035) and IL-10 decreased significantly (*P* = 0.041), none of the changes were significant after multiple testing adjustment. No significant associations or changes between groups were observed for any of the other molecular markers.

The results of linear models of change in MSEL receptive language raw score by changes in molecular measures in each group showed that there is a significant relationship between the pre-post change in MSEL receptive language raw score and change in several cytokines which were not statistically significant following adjustment for multiple testing.

## Discussion

Selective serotonin reuptake inhibitors, including sertraline, inhibit the serotonin transporter which normally reuptakes serotonin into presynaptic serotonergic neurons, subsequently, increasing extracellular levels of serotonin (Blakely et al., 1991). Sertraline has been approved to treat OCD in patients with ASD, based on the shared core symptoms of repetitive thoughts and behaviors in addition to the dysfunction of serotonergic system (Bastani et al., 1991; McDougle et al., 2000).

In this study we investigated the predictive efficacy of molecular biomarkers of sertraline, including *BDNF*, *MMP-9*, *TPH-2* and cytokines to determine whether sertraline normalizes the expression of any of these genes in young children with ASD.

*BDNF* is important for the regulation of neurodevelopment and neuroplasticity thus contributing to normal learning process and memory. Several studies demonstrated that *BDNF* levels in young children with ASD are higher than aged-match typical neurodevelopmental adults (Perry et al., 2001; Miyazaki et al., 2004). Excess of *BDNF* and other neurotrophins may affect tissue volume (Conover et al., 1995; Wassink et al., 1999). Taken together, elevated *BDNF* at early life may play an etiological role in ASD. This might be represented by the brain overgrowth as observed in many ASD children (Courchesne et al., 2001; Wassink et al., 2007; Lainhart and Lange, 2011; Hazlett et al., 2012; Armeanu et al., 2017). Consistently with previous studies we found that *BDNF* levels were significantly higher in ASD compared to controls (Miyazaki et al., 2004; Connolly et al., 2006; Nelson et al., 2006; Nishimura et al., 2007; Correia et al., 2010; Ricci et al., 2013; Zhang et al., 2014). However, these increased levels of *BDNF* were not normalized by sertraline. Specifically, our results show that the elevated *BDNF* levels in ASD are significantly larger than controls and sertraline can partially reduce the *BDNF* levels both in the treatment and placebo groups although there was no significantly difference after correction. As peripheral expression levels of *BDNF* in rats are associated with the level in CNS, we might imply that the concentration of peripheral *BDNF* can reflect the expression level in ASD brain as well (Karege et al., 2002; Fernandes et al., 2015). Accordingly, peripheral *BDNF* might be a potential biomarker for ASD (Zheng et al., 2016).

Plasma *MMP-9* expression levels in ASD were significantly lower than controls which is contrary to the elevated *MMP-9* levels in amniotic fluid samples from 331 ASD cases suggesting fluctuation of *MMP-9* during early development in ASD (Abdallah et al., 2012). Despite different *MMP-9* expression levels, sertraline had no effect on *MMP-9*. *MMP-9* plays a pivotal role in neuronal survival, CNS development, synaptic plasticity, and neuroinflammation through triggering several neurotrophic factors including *BDNF* (Gijbels et al., 1994; Rosenberg, 2002; Ethell and Ethell, 2007; Fujioka et al., 2012). Hence, it is plausible that *MMP-9* might contribute to the etiopathology of ASD as well. On the other hands, *MMP-9* can also mediate the inflammatory response through promoting proteolysis either by stimulating or suppressing inflammatory cytokines involved in ASD pathology (Yoo et al., 2002; Parks et al., 2004; Deverman and Patterson, 2009). However, in our study, no significant differences were observed.

Extensive evidence has suggested the role of pro-inflammatory and anti-inflammatory cytokines involving the severity of problematic behaviors and developmental and adaptive malfunctions as seen in ASD (Ashwood et al., 2011a, b, Napolioni et al., 2013). However, heterogeneity of immune function findings in ASD may imply that there is no definite type of inflammatory response responsible for the etiology of ASD (Mead and Ashwood, 2015).

The human *TPH2* is exclusively expressed in the brain, particularly in the serotonergic neurons of the dorsal and median raphe nuclei which is the primary source of serotonin (Walther et al., 2003; Bach-Mizrachi et al., 2006; Zill et al., 2007). Accumulating evidence has proposed that several functional polymorphisms of *TPH2* gene are associated with psychiatric disorders including major depressive disorder (MDD), attention deficit hyperactivity disorder (ADHD), schizophrenia, and bipolar disorder (De Luca et al., 2004; De Luca et al., 2005; Sheehan et al., 2005; Cichon et al., 2007; Haghighi et al., 2008; Gao et al., 2012).

The functional consequences of single nucleotide polymorphisms (SNPs) are not clear but could potentially modify the expression and the function of the *TPH2* gene. Candidate *TPH2* variants including the ones in our study have long been well replicated in case of MDD (Mandelli et al., 2012; Van der Auwera et al., 2014; Han et al., 2017). Further, associations between *TPH2* polymorphisms and ASD susceptibility particularly repetitive and stereotyped behaviors suggesting that they might affect the ASD phenotypes and augment ASD susceptibility have been reported (Coon et al., 2005; Yang et al., 2012; Barrie et al., 2018). However, in our treatment group the rs11178997 with AT genotype demonstrated significantly higher expression language raw scores than the TT genotype. Furthermore, variant rs4290270 with AA genotype showed slightly increased scores compared to TT genotype. Our findings suggest that sertraline might play a moderate role in expressive language in ASD depending on the *TPH2* genotype.

## Conclusion

In conclusion, sertraline had no distinct effect on young children with ASD compared to placebo and on several biomarkers including in this trial albeit their potential from ample previous studies. Future investigations should be of longer duration and should include more ASD subjects, which is a limitation of this study, with less heterogeneity to gain more insights on how to improve the quality of life of children with ASD.

## Data Availability Statement

The raw data supporting the conclusions of this article will be made available by the authors, without undue reservation, to any qualified researcher.

## Ethics Statement

The studies involving human participants were reviewed and approved by UC Davis Institutional Review Board. Written informed consent to participate in this study was provided by the participants’ legal guardian/next of kin.

## Author Contributions

RA conducted the experiments, participated to the discussion of the study, and wrote the manuscript. PJ participated to the discussion of the study and wrote the manuscript. BD-J performed the statistical analysis and participated to the writing of the manuscript. MJ conducted the experiments and participated to the writing of the manuscript. HT-T conducted the some experiments, help with the graphic, and revised the manuscript. RH provided the clinical assessment of the participants and revised the manuscript. FT designed the study and participated to the data analysis, and to the writing of the manuscript. All co-authors approved the final manuscript as it was submitted.

## Conflict of Interest

FT received funds from Asuragen, Inc., and Zynerba. RH has received funding from Zynerba, Ovid, and the Azrieli Foundation for carrying out treatment studies in patients with FXS. She has also consulted with Fulcrum, and Zynerba regarding treatment studies in the same population. The remaining authors declare that the research was conducted in the absence of any commercial or financial relationships that could be construed as a potential conflict of interest.

## References

[B1] Abdallah M. W. Pearce B. D. Larsen N. Greaves-Lord K. Norgaard-Pedersen B. Hougaard D. M. (2012). Amniotic fluid MMP-9 and neurotrophins in autism spectrum disorders: an exploratory study. *Autism Res.* 5 428–433. 10.1002/aur.1254 23008271

[B2] Agresti A. Kateri M. (2011). “Categorical data analysis,” in *International Encyclopedia of Statistical Science*, ed. Lovric M. (Berlin: Springer), 206–208.

[B3] Anderson G. M. Horne W. C. Chatterjee D. Cohen D. J. (1990). The hyperserotonemia of autism. *Ann. N. Y. Acad. Sci.* 600 331–340. 2252319 10.1111/j.1749-6632.1990.tb16893.x

[B4] Anderson G. M. Lombroso P. J. (2002). Genetics of Childhood Disorders: XLV. Autism Part 4: serotonin in Autism. *J. Am. Acad. Child Adolesc. Psychiatry* 41 1513–1516. 10.1097/00004583-200212000-00025 12447040

[B5] APA (ed.) (2013). *Diagnostic and Statistical Manual of Mental Disorders.* Washington, DC: APA.

[B6] Armeanu R. Mokkonen M. Crespi B. (2017). Meta-Analysis of BDNF Levels in Autism. *Cell. Mol. Neurobiol.* 37 949–954. 10.1007/s10571-016-0415-7 27501933 PMC11482231

[B7] Ashwood P. Krakowiak P. Hertz-Picciotto I. Hansen R. Pessah I. Van de Water J. (2011a). Elevated plasma cytokines in autism spectrum disorders provide evidence of immune dysfunction and are associated with impaired behavioral outcome. *Brain Behav. Immun.* 25 40–45. 10.1016/j.bbi.2010.08.003 20705131 PMC2991432

[B8] Ashwood P. Krakowiak P. Hertz-Picciotto I. Hansen R. Pessah I. N. Van de Water J. (2011b). Associations of impaired behaviors with elevated plasma chemokines in autism spectrum disorders. *J. Neuroimmunol.* 232 196–199. 10.1016/j.jneuroim.2010.10.025 21095018 PMC3053074

[B9] Bach-Mizrachi H. Underwood M. D. Kassir S. A. Bakalian M. J. Sibille E. Tamir H. (2006). Neuronal tryptophan hydroxylase mRNA expression in the human dorsal and median raphe nuclei: major depression and suicide. *Neuropsychopharmacology* 31 814–824. 10.1038/sj.npp.1300897 16192985

[B10] Barrie E. S. Pinsonneault J. K. Sadee W. Hollway J. A. Handen B. L. Smith T. (2018). Testing genetic modifiers of behavior and response to atomoxetine in autism spectrum disorder with ADHD. *J. Dev. Phys. Disabil.* 30 355–371. 10.1007/s10882-018-9590-4 30197492 PMC6128165

[B11] Bastani B. Arora R. C. Meltzer H. Y. (1991). Serotonin uptake and imipramine binding in the blood platelets of obsessive-compulsive disorder patients. *Biol. Psychiatry* 30 131–139. 10.1016/0006-3223(91)90166-j 1655071

[B12] Benjamini Y. Hochberg Y. (1995). Controlling the false discovery rate: a practical and powerful approach to multiple testing. *J. R. Stat. Soc. Ser. B* 57 289–300. 10.1111/j.2517-6161.1995.tb02031.x

[B13] Binder D. K. Scharfman H. E. (2004a). Brain-derived neurotrophic factor. *Growth Factors* 22 123–131. 15518235 10.1080/08977190410001723308 PMC2504526

[B14] Binder D. K. Scharfman H. E. (2004b). Mini Review. *Growth Factors* 22 123–131. 15518235 10.1080/08977190410001723308 PMC2504526

[B15] Blakely R. D. Berson H. E. Fremeau R. T. Jr. Caron M. G. Peek M. M. (1991). Cloning and expression of a functional serotonin transporter from rat brain. *Nature* 354 66–70. 10.1038/354066a0 1944572

[B16] Brooks-Kayal A. (2010). Epilepsy and autism spectrum disorders: are there common developmental mechanisms?. *Brain Dev.* 32 731–738. 10.1016/j.braindev.2010.04.010 20570072

[B17] Busner J. Targum S. D. (2007). The clinical global impressions scale: applying a research tool in clinical practice. *Psychiatry* 4 28–37. PMC2880930 20526405

[B18] Butler M. G. Youngs E. L. Roberts J. L. Hellings J. A. (2012). Assessment and treatment in autism spectrum disorders: a focus on genetics and psychiatry. *Autism Res. Treat.* 2012:242537. 10.1155/2012/242537 PMC3420490 22934170

[B19] Cadoret R. J. Langbehn D. Caspers K. Troughton E. P. Yucuis R. Sandhu H. K. (2003). Associations of the serotonin transporter promoter polymorphism with aggressivity, attention deficit, and conduct disorder in an adoptee population. *Compr. Psychiatry* 44 88–101. 10.1053/comp.2003.50018 12658617

[B20] Chandana S. R. Behen M. E. Juhasz C. Muzik O. Rothermel R. D. Mangner T. J. (2005). Significance of abnormalities in developmental trajectory and asymmetry of cortical serotonin synthesis in autism. *Int. J. Dev. Neurosci.* 23 171–182. 10.1016/j.ijdevneu.2004.08.002 15749243

[B21] Christensen R. H. B. (2019). *ordinal - Regression Models for Ordinal Data. R package version* 2019.4-25. Available online at: https://github.com/runehaubo/ordinal

[B22] Chugani D. C. (2002). Role of altered brain serotonin mechanisms in autism. *Mol. Psychiatry* 7(Suppl. 2), S16–S17. 12142936 10.1038/sj.mp.4001167

[B23] Chugani D. C. (2012). Neuroimaging and neurochemistry of autism. *Pediatr. Clin. North Am.* 59 63–73, x. 22284793 10.1016/j.pcl.2011.10.002

[B24] Chugani D. C. Muzik O. Behen M. Rothermel R. Janisse J. J. Lee J. (1999). Developmental changes in brain serotonin synthesis capacity in autistic and nonautistic children. *Ann. Neurol.* 45 287–295. 10.1002/1531-8249(199903)45:3<287::aid-ana3>3.0.co;2-9 10072042

[B25] Cichon S. Winge I. Mattheisen M. Georgi A. Karpushova A. Freudenberg J. (2007). Brain-specific tryptophan hydroxylase 2 (TPH2): a functional Pro206Ser substitution and variation in the 5′-region are associated with bipolar affective disorder. *Hum. Mol. Genet.* 17 87–97. 10.1093/hmg/ddm286 17905754

[B26] Connolly A. M. Chez M. Streif E. M. Keeling R. M. Golumbek P. T. (2006). Brain-derived neurotrophic factor and autoantibodies to neural antigens in sera of children with autistic spectrum disorders, Landau-Kleffner syndrome, and epilepsy. *Biol. Psychiatry* 59 354–363. 10.1016/j.biopsych.2005.07.004 16181614

[B27] Conover J. C. Erickson J. T. Katz D. M. Bianchi L. M. Poueymirou W. T. McClain J. (1995). Neuronal deficits, not involving motor neurons, in mice lacking BDNF and/or NT4. *Nature* 375 235–238. 10.1038/375235a0 7746324

[B28] Cook E. H. Leventhal B. L. (1996). The serotonin system in autism. *Curr. Opin. Pediatr.* 8 348–354. 10.1097/00008480-199608000-00008 9053096

[B29] Coon H. Dunn D. Lainhart J. Miller J. Hamil C. Battaglia A. (2005). Possible association between autism and variants in the brain-expressed tryptophan hydroxylase gene (TPH2). *Am. J. Med. Genet. B Neuropsychiatr. Genet.* 135b 42–46. 10.1002/ajmg.b.30168 15768392

[B30] Correia C. T. Coutinho A. M. Sequeira A. F. Sousa I. G. Lourenço Venda L. (2010). Increased BDNF levels and NTRK2 gene association suggest a disruption of BDNF/TrkB signaling in autism. *Genes Brain Behav.* 9 841–848. 10.1111/j.1601-183x.2010.00627.x 20662941

[B31] Courchesne E. Carper R. Akshoomoff N. (2003). Evidence of brain overgrowth in the first year of life in autism. *JAMA* 290 337–344. 12865374 10.1001/jama.290.3.337

[B32] Courchesne E. Karns C. M. Davis H. R. Ziccardi R. Carper R. A. Tigue Z. D. (2001). Unusual brain growth patterns in early life in patients with autistic disorder: an MRI study. *Neurology* 57 245–254. 10.1212/wnl.57.2.245 11468308

[B33] Daws L. C. Munn J. L. Valdez M. F. Frosto-Burke T. Hensler J. G. (2007). Serotonin transporter function, but not expression, is dependent on brain-derived neurotrophic factor (BDNF): *in vivo* studies in BDNF-deficient mice. *J. Neurochem.* 101 641–651. 10.1111/j.1471-4159.2006.04392.x 17254018

[B34] De Luca V. Mueller D. J. Tharmalingam S. King N. Kennedy J. L. (2004). Analysis of the novel TPH2 gene in bipolar disorder and suicidality. *Mol. Psychiatry* 9 896–897. 10.1038/sj.mp.4001531 15197398

[B35] De Luca V. Voineskos D. Wong G. W. Shinkai T. Rothe C. Strauss J. (2005). Promoter polymorphism of second tryptophan hydroxylase isoform (TPH2) in schizophrenia and suicidality. *Psychiatry Res.* 134 195–198. 10.1016/j.psychres.2005.01.005 15840421

[B36] Deverman B. E. Patterson P. H. (2009). Cytokines and CNS development. *Neuron* 64 61–78. 10.1016/j.neuron.2009.09.002 19840550

[B37] Dityatev A. Schachner M. Sonderegger P. (2010). The dual role of the extracellular matrix in synaptic plasticity and homeostasis. *Nat. Rev. Neurosci.* 11 735–746. 10.1038/nrn2898 20944663

[B38] Ethell I. M. Ethell D. W. (2007). Matrix metalloproteinases in brain development and remodeling: synaptic functions and targets. *J. Neurosci. Res.* 85 2813–2823. 10.1002/jnr.21273 17387691

[B39] Fernandes B. S. Steiner J. Berk M. Molendijk M. L. Gonzalez-Pinto A. Turck C. W. (2015). Peripheral brain-derived neurotrophic factor in schizophrenia and the role of antipsychotics: meta-analysis and implications. *Mol. Psychiatry* 20 1108–1119. 10.1038/mp.2014.117 25266124

[B40] Fujioka H. Dairyo Y. Yasunaga K. Emoto K. (2012). Neural functions of matrix metalloproteinases: plasticity, neurogenesis, and disease. *Biochem Res. Int.* 2012:789083. 10.1155/2012/789083 PMC3332068 22567285

[B41] Gao J. Pan Z. Jiao Z. Li F. Zhao G. Wei Q. (2012). TPH2 gene polymorphisms and major depression–a meta-analysis. *PLoS One* 7:e36721. 10.1371/journal.pone.0036721 22693556 PMC3365065

[B42] Gijbels K. Galardy R. E. Steinman L. (1994). Reversal of experimental autoimmune encephalomyelitis with a hydroxamate inhibitor of matrix metalloproteases. *J. Clin. Invest.* 94 2177–2182. 10.1172/jci117578 7989572 PMC330042

[B43] Goldberg J. Anderson G. M. Zwaigenbaum L. Hall G. B. Nahmias C. Thompson A. (2009). Cortical serotonin type-2 receptor density in parents of children with autism spectrum disorders. *J. Autism. Dev. Disord.* 39 97–104. 10.1007/s10803-008-0604-4 18592367

[B44] Gunstad J. Benitez A. Smith J. Glickman E. Spitznagel M. B. Alexander T. (2008). Serum brain-derived neurotrophic factor is associated with cognitive function in healthy older adults. *J. Geriatr. Psychiatry Neurol.* 21 166–170. 10.1177/0891988708316860 18503034

[B45] Haghighi F. Bach-Mizrachi H. Huang Y. Y. Arango V. Shi S. Dwork A. J. (2008). Genetic architecture of the human tryptophan hydroxylase 2 Gene: existence of neural isoforms and relevance for major depression. *Mol. Psychiatry* 13 813–820. 10.1038/sj.mp.4002127 18180764

[B46] Han K. M. Won E. Kang J. Kim A. Yoon H. K. Chang H. S. (2017). Local gyrification index in patients with major depressive disorder and its association with tryptophan hydroxylase-2 (TPH2) polymorphism. *Hum. Brain Mapp.* 38 1299–1310. 10.1002/hbm.23455 27807918 PMC6866875

[B47] Hazlett H. C. Poe M. D. Lightbody A. A. Styner M. MacFall J. R. Reiss A. L. (2012). Trajectories of early brain volume development in fragile X syndrome and autism. *J. Am. Acad. Child Adolesc. Psychiatry* 51 921–933. 10.1016/j.jaac.2012.07.003 22917205 PMC3428739

[B48] Hranilovic D. Bujas-Petkovic Z. Vragovic R. Vuk T. Hock K. Jernej B. (2007). Hyperserotonemia in adults with autistic disorder. *J. Autism Dev. Disord.* 37 1934–1940. 10.1007/s10803-006-0324-6 17165147

[B49] Huntley G. W. (2012). Synaptic circuit remodelling by matrix metalloproteinases in health and disease. *Nat. Rev. Neurosci.* 13 743–757. 10.1038/nrn3320 23047773 PMC4900464

[B50] Karege F. Schwald M. Cisse M. (2002). Postnatal developmental profile of brain-derived neurotrophic factor in rat brain and platelets. *Neurosci. Lett.* 328 261–264. 10.1016/s0304-3940(02)00529-3 12147321

[B51] Kolevzon A. Mathewson K. A. Hollander E. (2006). Selective serotonin reuptake inhibitors in autism: a review of efficacy and tolerability. *J. Clin. Psychiatry* 67 407–414. 10.4088/jcp.v67n0311 16649827

[B52] Lainhart J. E. Bigler E. D. Bocian M. Coon H. Dinh E. Dawson G. (2006). Head circumference and height in autism: a study by the Collaborative Program of Excellence in Autism. *Am. J. Med. Genet. A* 140 2257–2274. 10.1002/ajmg.a.31465 17022081 PMC4899843

[B53] Lainhart J. E. Lange N. (2011). Increased neuron number and head size in autism. *JAMA* 306 2031–2032. 22068999 10.1001/jama.2011.1633

[B54] Lecavalier L. (2006). Behavioral and emotional problems in young people with pervasive developmental disorders: relative prevalence, effects of subject characteristics, and empirical classification. *J. Autism Dev. Disord.* 36 1101–1114. 10.1007/s10803-006-0147-5 16897387

[B55] Lord C. Elsabbagh M. Baird G. Veenstra-Vanderweele J. (2018). Autism spectrum disorder. *Lancet* 392 508–520. 30078460 10.1016/S0140-6736(18)31129-2 PMC7398158

[B56] Makkonen I. Riikonen R. Kokki H. Airaksinen M. M. Kuikka J. T. (2008). Serotonin and dopamine transporter binding in children with autism determined by SPECT. *Dev. Med. Child Neurol.* 50 593–597. 10.1111/j.1469-8749.2008.03027.x 18754896

[B57] Mandelli L. Antypa N. Nearchou F. A. Vaiopoulos C. Stefanis C. N. Serretti A. (2012). The role of serotonergic genes and environmental stress on the development of depressive symptoms and neuroticism. *J. Affect. Disord.* 142 82–89. 10.1016/j.jad.2012.03.047 22868061

[B58] Marazziti D. Muratori F. Cesari A. Masala I. Baroni S. Giannaccini G. (2000). Increased density of the platelet serotonin transporter in autism. *Pharmacopsychiatry* 33 165–168. 10.1055/s-2000-7588 11071017

[B59] Mattson M. P. Maudsley S. Martin B. (2004). BDNF and 5-HT: a dynamic duo in age-related neuronal plasticity and neurodegenerative disorders. *Trends Neurosci.* 27 589–594. 10.1016/j.tins.2004.08.001 15374669

[B60] McAllister A. K. (2001). Neurotrophins and neuronal differentiation in the central nervous system. *Cell. Mol. Life Sci.* 58 1054–1060. 10.1007/pl00000920 11529498 PMC11337387

[B61] McDougle C. J. Brodkin E. S. Naylor S. T. Carlson D. C. Cohen D. J. Price L. H. (1998). Sertraline in adults with pervasive developmental disorders: a prospective open-label investigation. *J. Clin. Psychopharmacol.* 18 62–66. 10.1097/00004714-199802000-00010 9472844

[B62] McDougle C. J. Kresch L. E. Posey D. J. (2000). Repetitive thoughts and behavior in pervasive developmental disorders: treatment with serotonin reuptake inhibitors. *J. Autism Dev. Disord.* 30 427–435. 11098879 10.1023/a:1005551523657

[B63] McKinney J. Knappskog P. M. Haavik J. (2005). Different properties of the central and peripheral forms of human tryptophan hydroxylase. *J. Neurochem.* 92 311–320. 10.1111/j.1471-4159.2004.02850.x 15663479

[B64] Mead J. Ashwood P. (2015). Evidence supporting an altered immune response in ASD. *Immunol. Lett.* 163 49–55. 10.1016/j.imlet.2014.11.006 25448709

[B65] Meng W. D. Sun S. J. Yang J. Chu R. X. Tu W. Liu Q. (2017). Elevated serum brain-derived neurotrophic factor (BDNF) but not BDNF Gene Val66Met polymorphism is associated with autism spectrum disorders. *Mol. Neurobiol.* 54 1167–1172. 10.1007/s12035-016-9721-9 26820673

[B66] Miles J. H. Hillman R. E. (2000). Value of a clinical morphology examination in autism. *Am. J. Med. Genet.* 91 245–253. 10.1002/(sici)1096-8628(20000410)91:4<245::aid-ajmg1>3.0.co;2-2 10766977

[B67] Miyazaki K. Narita N. Sakuta R. Miyahara T. Naruse H. Okado N. (2004). Serum neurotrophin concentrations in autism and mental retardation: a pilot study. *Brain Dev.* 26 292–295. 10.1016/s0387-7604(03)00168-2 15165668

[B68] Mulder E. J. (2006). *The Hyperserotonemia of Autism Spectrum Disorders.* Groningen: University Library Groningen.

[B69] Mullen E. (1995). *Mullen Scales of Early Learning (AGS Edition).* Circle Pines, MN: American Guidance Services, Inc.

[B70] Nadeau J. Sulkowski M. L. Ung D. Wood J. J. Lewin A. B. Murphy T. K. (2011). Treatment of comorbid anxiety and autism spectrum disorders. *Neuropsychiatry* 1 567–578. 10.2217/npy.11.62 24174992 PMC3809000

[B71] Nakamura K. Sekine Y. Ouchi Y. Tsujii M. Yoshikawa E. Futatsubashi M. (2010). Brain serotonin and dopamine transporter bindings in adults with high-functioning autism. *Arch. Gen. Psychiatry* 67 59–68. 20048223 10.1001/archgenpsychiatry.2009.137

[B72] Napolioni V. Ober-Reynolds B. Szelinger S. Corneveaux J. J. Pawlowski T. Ober-Reynolds S. (2013). Plasma cytokine profiling in sibling pairs discordant for autism spectrum disorder. *J. Neuroinflammation* 10:38. 10.1186/1742-2094-10-38 PMC3616926 23497090

[B73] Nelson P. G. Kuddo T. Song E. Y. Dambrosia J. M. Kohler S. (2006). Selected neurotrophins, neuropeptides, and cytokines: developmental trajectory and concentrations in neonatal blood of children with autism or Down syndrome. *Int. J. Dev. Neurosci.* 24 73–80. 10.1016/j.ijdevneu.2005.10.003 16289943

[B74] Nishimura K. Nakamura K. Anitha A. Yamada K. Tsujii M. Iwayama Y. (2007). Genetic analyses of the brain-derived neurotrophic factor (BDNF) gene in autism. *Biochem. Biophys. Res. Commun.* 356 200–206. 17349978 10.1016/j.bbrc.2007.02.135

[B75] Noroozi R. Taheri M. Movafagh A. Mirfakhraie R. Solgi G. Sayad A. (2016). Glutamate receptor, metabotropic 7 (GRM7) gene variations and susceptibility to autism: a case–control study. *Autism Res.* 9 1161–1168. 10.1002/aur.1640 27312574

[B76] Oblak A. Gibbs T. T. Blatt G. J. (2013). Reduced serotonin receptor subtypes in a limbic and a neocortical region in autism. *Autism Res.* 6 571–583. 10.1002/aur.1317 23894004 PMC3859849

[B77] Parks W. C. Wilson C. L. López-Boado Y. S. (2004). Matrix metalloproteinases as modulators of inflammation and innate immunity. *Nat. Rev. Immunol.* 4 617–629. 10.1038/nri1418 15286728

[B78] Perry E. K. Lee M. L. Martin-Ruiz C. M. Court J. A. Volsen S. G. Merrit J. (2001). Cholinergic activity in autism: abnormalities in the cerebral cortex and basal forebrain. *Am. J. Psychiatry* 158 1058–1066. 10.1176/appi.ajp.158.7.1058 11431227

[B79] Potter L. A. Scholze D. A. Biag H. M. B. Schneider A. Chen Y. Nguyen D. V. (2019). A randomized controlled trial of sertraline in young children with autism spectrum disorder. *Front. Psychiatry* 10:810. 10.3389/fpsyt.2019.00810 31780970 PMC6851992

[B80] R Core Team (2019). *R: A Language and Environment for Statistical Computing.* Vienna: R Foundation for Statistical Computing.

[B81] Ramoz N. Cai G. Reichert J. G. Corwin T. E. Kryzak L. A. Smith C. J. (2006). Family-based association study of TPH1 and TPH2 polymorphisms in autism. *Am. J. Med. Genet. B Neuropsychiatr. Genet.* 141b 861–867. 10.1002/ajmg.b.30356 16958027

[B82] Ricci S. Businaro R. Ippoliti F. Lo Vasco V. R. Massoni F. Onofri E. (2013). Altered cytokine and BDNF levels in autism spectrum disorder. *Neurotox. Res.* 24 491–501. 10.1007/s12640-013-9393-4 23604965

[B83] Rosenberg G. A. (2002). Matrix metalloproteinases in neuroinflammation. *Glia* 39 279–291. 10.1002/glia.10108 12203394

[B84] Sacco R. Papaleo V. Hager J. Rousseau F. Moessner R. Militerni R. (2007). Case-control and family-based association studies of candidate genes in autistic disorder and its endophenotypes: TPH2 and GLO1. *BMC Med. Genet.* 8:11. 10.1186/1471-2350-8-11 17346350 PMC1851007

[B85] Sheehan K. Lowe N. Kirley A. Mullins C. Fitzgerald M. Gill M. (2005). Tryptophan hydroxylase 2 (TPH2) gene variants associated with ADHD. *Mol. Psychiatry* 10 944–949. 10.1038/sj.mp.4001698 15940290

[B86] Steingard R. J. Zimnitzky B. DeMaso D. R. Bauman M. L. Bucci J. P. (1997). Sertraline treatment of transition-associated anxiety and agitation in children with autistic disorder. *J. Child Adolesc. Psychopharmacol.* 7 9–15. 10.1089/cap.1997.7.9 9192538

[B87] Tassone F. Qi L. Zhang W. Hansen R. L. Pessah I. N. Hertz-Picciotto I. (2011). MAOA, DBH, and SLC6A4 variants in CHARGE: a case-control study of autism spectrum disorders. *Autism Res.* 4 250–261. 10.1002/aur.196 21538940 PMC3151322

[B88] Udo H. Jin I. Kim J.-H. Li H.-L. Youn T. Hawkins R. D. (2005). Serotonin-Induced Regulation of the Actin Network for Learning-Related Synaptic Growth Requires Cdc42, N-WASP, and PAK in Aplysia Sensory Neurons. *Neuron* 45 887–901. 10.1016/j.neuron.2005.01.044 15797550

[B89] Van der Auwera S. Janowitz D. Schulz A. Homuth G. Nauck M. Volzke H. (2014). Interaction among childhood trauma and functional polymorphisms in the serotonin pathway moderate the risk of depressive disorders. *Eur. Arch. Psychiatry Clin. Neurosci.* 264(Suppl. 1), S45–S54. 25214390 10.1007/s00406-014-0536-2

[B90] Walther D. J. Peter J. U. Bashammakh S. Hortnagl H. Voits M. Fink H. (2003). Synthesis of serotonin by a second tryptophan hydroxylase isoform. *Science* 299:76. 10.1126/science.1078197 12511643

[B91] Wassink T. H. Hazlett H. C. Epping E. A. Arndt S. Dager S. R. Schellenberg G. D. (2007). Cerebral cortical gray matter overgrowth and functional variation of the serotonin transporter gene in autism. *Arch. Gen. Psychiatry* 64 709–717. 17548752 10.1001/archpsyc.64.6.709

[B92] Wassink T. H. Nelson J. J. Crowe R. R. Andreasen N. C. (1999). Heritability of BDNF alleles and their effect on brain morphology in schizophrenia. *Am. J. Med. Genet. Neuropsychiatr. Genet.* 88 724–728. 10.1002/(sici)1096-8628(19991215)88:6<724::aid-ajmg25>3.0.co;2-7 10581496

[B93] Yang S. Y. Yoo H. J. Cho I. H. Park M. Kim S. A. (2012). Association with tryptophan hydroxylase 2 gene polymorphisms and autism spectrum disorders in Korean families. *Neurosci. Res.* 73 333–336. 10.1016/j.neures.2012.05.012 22698779

[B94] Yong V. W. (2005). Metalloproteinases: mediators of pathology and regeneration in the CNS. *Nat. Rev. Neurosci.* 6 931–944. 10.1038/nrn1807 16288297

[B95] Yoo H. G. Shin B. A. Park J. S. Lee K. H. Chay K. O. Yang S. Y. (2002). IL-1beta induces MMP-9 via reactive oxygen species and NF-kappaB in murine macrophage RAW 264.7 cells. *Biochem. Biophys. Res. Commun.* 298 251–256. 10.1016/s0006-291x(02)02431-2 12387824

[B96] Yoo M. H. Kim Y. T. Yoon Y. H. Koh J.-Y. (2016). Autism phenotypes in ZnT3 null mice: Involvement of zinc dyshomeostasis, MMP-9 activation and BDNF upregulation. *Sci. Rep.* 6:28548. 10.1038/srep28548 PMC4926223 27352957

[B97] Zafeiriou D. I. Ververi A. Vargiami E. (2009). The serotonergic system: its role in pathogenesis and early developmental treatment of autism. *Curr. Neuropharmacol.* 7 150–157. 10.2174/157015909788848848 19949574 PMC2730007

[B98] Zhang Q.-B. Jiang L.-F. Kong L.-Y. Lu Y.-J. (2014). Serum Brain-derived neurotrophic factor levels in Chinese children with autism spectrum disorders: a pilot study. *Int. J. Dev. Neurosci.* 37 65–68. 10.1016/j.ijdevneu.2014.06.013 24984148

[B99] Zheng Z. Zhang L. Zhu T. Huang J. Qu Y. Mu D. (2016). Peripheral brain-derived neurotrophic factor in autism spectrum disorder: a systematic review and meta-analysis. *Sci. Rep.* 6:31241. 10.1038/srep31241 PMC4979025 27506602

[B100] Zill P. Buttner A. Eisenmenger W. Moller H. J. Ackenheil M. Bondy B. (2007). Analysis of tryptophan hydroxylase I and II mRNA expression in the human brain: a post-mortem study. *J. Psychiatr. Res.* 41 168–173. 10.1016/j.jpsychires.2005.05.004 16023677

